# Dystonia and the pedunculopontine nucleus: Current evidences and potential mechanisms

**DOI:** 10.3389/fneur.2022.1065163

**Published:** 2022-11-23

**Authors:** Jun-hui Su, Yao-wen Hu, Yi Yang, Ruo-yu Li, Fei Teng, Li-xi Li, Ling-jing Jin

**Affiliations:** ^1^Department of Neurology, Shanghai Tongji Hospital, School of Medicine, Tongji University, Shanghai, China; ^2^Department of Neurology and Neurological Rehabilitation, Shanghai YangZhi Rehabilitation Hospital (Shanghai Sunshine Rehabilitation Center), School of Medicine, Tongji University, Shanghai, China

**Keywords:** pedunculopontine nucleus, dystonia, neuropathology, muscle tone, deep brain stimulation

## Abstract

Being a major component of the midbrain locomotion region, the pedunculopontine nucleus (PPN) is known to have various connections with the basal ganglia, the cerebral cortex, thalamus, and motor regions of the brainstem and spinal cord. Functionally, the PPN is associated with muscle tone control and locomotion modulation, including motor initiation, rhythm and speed. In addition to its motor functions, the PPN also contribute to level of arousal, attention, memory and learning. Recent studies have revealed neuropathologic deficits in the PPN in both patients and animal models of dystonia, and deep brain stimulation of the PPN also showed alleviation of axial dystonia in patients of Parkinson's disease. These findings indicate that the PPN might play an important role in the development of dystonia. Moreover, with increasing preclinical evidences showed presence of dystonia-like behaviors, muscle tone changes, impaired cognitive functions and sleep following lesion or neuromodulation of the PPN, it is assumed that the pathological changes of the PPN might contribute to both motor and non-motor manifestations of dystonia. In this review, we aim to summarize the involvement of the PPN in dystonia based on the current preclinical and clinical evidences. Moreover, potential mechanisms for its contributions to the manifestation of dystonia is also discussed base on the dystonia-related basal ganglia-cerebello-thalamo-cortical circuit, providing fundamental insight into the targeting of the PPN for the treatment of dystonia in the future.

## Introduction

The pedunculopontine nucleus (PPN) is located in the dorsal pontomesencephalic tegmentum, and it has wide anatomical connections with the central nervous system (1). Together with the cuneiform nucleus, the PPN is involved in the control of locomotion, including motor initiation, rhythm, and speed (2–4). In addition, the neural activity of the PPN has been reported closely associated with muscle tone control, along with its contributions to the level of arousal, attention, memory and learning (5, 6). Dystonia is a movement disorder characterized by abnormal involuntary movements or postures caused by sustained or intermittent muscle contractions, which are often repetitive (7). Pathologic changes of the PPN have been reported in patients of different types of dystonia, and the manifestation of dystonia could be alleviated through DBS of the PPN, reflecting a crucial role of the PPN in the development of dystonia (8–11). So far, increasing preclinical evidences also showed alterations of the muscle tone, locomotion, cognitive functions and sleep following lesion or neuromodulation of the PPN, which mimicked the motor and non-motor manifestations of dystonia (2, 5, 12–14). Moreover, from the anatomical aspects, the PPN has dense connections with the dystonia-related basal ganglia-cerebello-thalamo-cortical circuit, which lays the foundation of the PPN involved in dystonia (15).

## Anatomical and functional characteristics of the PPN

The PPN has been identified to be a major component of the mesencephalic locomotion region located in the brainstem. It has been divided into the rostral and caudal part which contain cholinergic, glutamatergic and GABAergic neurons (16). The cholinergic neurons are intermingled with numerous glutamatergic and GABAergic neurons, which are distributed throughout the rostro-caudal extent of the PPN. The GABAergic neurons are mainly located in the rostral part, whereas the glutamatergic neurons are abundantly situated in the caudal part (17). In addition, less than 5% of cholinergic neurons co-release either glutamate or GABA and may possess more complex motor functions (18).

The PPN is surrounded by the superior cerebellar peduncle (medially), the medial lemniscus (laterally), the red nucleus (rostrally), and the laterodorsal tegmental nucleus (LDT, caudally) (1). It has ascending and descending connections with multiple motor areas, including the motor and premotor cortex, basal ganglia, reticular formation, superior colliculus and cranial nerve nuclei (V, VII and XII) (6). The ascending network is involved in motor planning, selection, and sensory motor integration, whereas the descending network functions to directly modulate locomotion and muscle tone (19, 20). In the ascending motor connectome, the PPN has complex anatomical connections with the basal ganglia, which is known to play a crucial role in the central selection of competing alternative actions through direct, indirect, and hyperdirect pathways (21). In particular, GABAergic input from the internal part of the globus pallidus internus (GPi) and the substantia nigra pars reticulata (SNpr) and glutamatergic input from the subthalamic nucleus (STN) primarily project to the PPN (22, 23). Reciprocally, The SNpc and STN are densely innervated by the PPN. Cholinergic and glutamatergic neurons of the PPN form synaptic connections with dopaminergic neurons in the SNpc, which thereafter modulate the dopamine release in the striatum (24, 25). And both glutamatergic and GABAergic fibers project to the STN from the PPN to influence the indirect pathway of excessive movement inhibition (26). Additionally, a recent study reported that the cholinergic and glutamatergic neurons in the PPN could directly project to the striatum and form synaptic connections with medium-sized spiny neurons, cholinergic interneurons, and GABAergic interneurons and modulate their neural activity (19, 27). Of note, the PPN and LDT has been determined as the only external source of cholinergic projection of the striatum (28). Along with the basal ganglia, the PPN could also send cholinergic projection to the cerebellum and activate the deep cerebellar nuclei, while the cerebellum sends reciprocal projections to both cholinergic and glutamatergic neurons in the PPN (2, 29, 30). Since the cerebellum has a vital role in flexible modification of behavior and error-based learning, the PPN acts as an important interface between the cerebellum and basal ganglia which participates in the motor control and cognitive functions (31).

**Figure 1:**
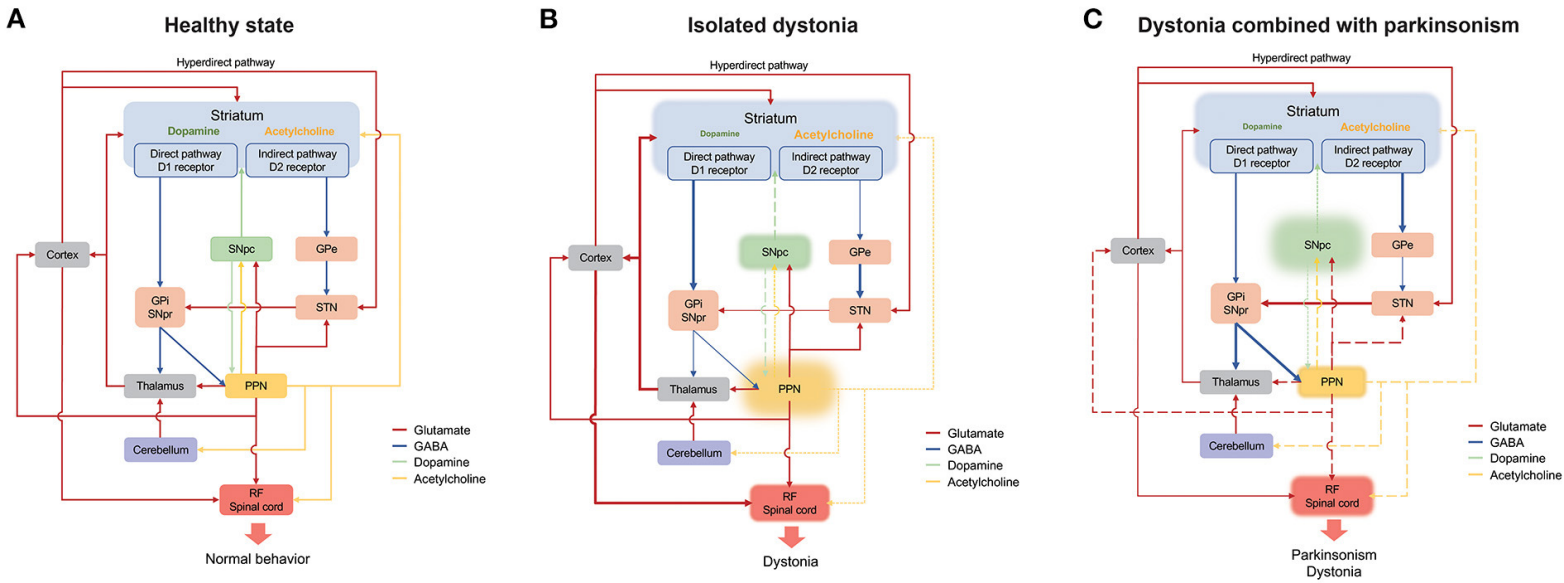
Schematic representation of the connection of the PPN in the basal ganglia-cerebello-thalamo-cortical circuit under normal **(A)**, isolated dystonia **(B)**, and dystonia combined with parkinsonism **(C)**. Thickened and thinned lines represent enhanced and decreased activity, respectively. Dashed lines show primarily loss whereas long dashed lines indicate secondarily loss. Cortico-striatum inputs modulate central selection of competing alternative actions through direct, indirect and hyperdirect pathways. And the inhibitory GABA outputs from GPi and SNpr project to the cortex through the thalamus and eventually participate in motor execution. The PPN has connections with the basal ganglia, the thalamus, cerebellum, and RF/spinal cord, with its cholinergic outputs mainly project to the striatum, the SNpc and the RF/spinal cord. In normal state **(A)**, adequate motor selection of the basal ganglia and the balance of the dopamine and the acetylcholine result in normal behavior. In isolated dystonia **(B)**, the PPN is primarily affected, which mainly involves the loss of cholinergic neurons. The cholinergic deficit of the PPN directly influences the neural activity of the striatum and the RF/spinal cord and indirectly affects the dopamine release in the striatum though SNpc, which result in the disturbance of central selection and the presence of dystonia. In dystonia combined with parkinsonism **(C)**, the dopaminergic system of the SNpc is primarily affected. The deficit of the dopamine release in the striatum results in the overactivity of the indirect pathway, which leads to the presence of parkinsonism. On the other hand, the pathology of the SNpc contributes to the dysfunction of both glutamatergic and cholinergic systems in the PPN, which thereafter directly induces the disinhibition of the RF/spinal cord or indirectly affects the function of the striatum, thalamus and cortex. GABA, γ-aminobutyric acid; GPe, globus pallidus externus; GPi, globus pallidus internus; PPN, pedunculopontine tegmental nucleus; RF, reticular formation; SNpc, substantia nigra pars compact; SNpc, substantia nigra pars reticulata (SNpr); STN, subthalamic nucleus.

Multiple cortex areas including the motor cortex, pre-motor cortex, somatosensory motor cortex and frontal eye fields send excitatory outputs to the PPN, and the PPN densely innervates the motor cortex, pre-motor cortex and frontal lope, respectively. The PPN might affect the cortical functions of movements, cognitive and sleep through these connections (32). With regard to the thalamus, the PPN can send strong cholinergic projections to the thalamic nuclei, and the ascending reticular activating system connects to the cortex via these cholinergic projections of the PPN to the thalamus, which is involved in the arousal and rapid eye movement sleep (32). In addition, the PPN can send cholinergic output to the thalamic centromedian-parafascicular complex, which in turn connects to multiple cortex areas and allows the PPN participating in modulation of action selection, attention, memory, learning, spatial perception, impulsivity control and decision making (6). Other brain areas, such as the superior and inferior colliculi and periaqueductal gray, also have reciprocal projections with the PPN (4). Meanwhile, descending fibers from the PPN directly feed to the pontine and medullary reticular formation and spinal cord, including the pontine reticular nuclei oralis and caudalis and the gigantocellular reticular nucleus, which might participate in the locomotion and muscle tone control (5, 6) (Figure 1A).

## Preclinical findings of the involvement of the PPN in dystonia

In preclinical experiments, the relationship between the PPN and dystonia was primarily revealed through the neurochemical and pathological findings in dystonia animal models. Additionally, stereotaxic modulation of the PPN in normal animals also provided evidence of its effect on muscle tone and dystonia manifestation.

### Pathologic changes of the PPN in animal models of dystonia

The cholinergic pathology of the PPN was observed in genotypic animal models of dystonia (33, 34). As for mouse models of Dyt1/Tor1a gene mutation, including the Dyt1 knock-in, knockdown, and knockout model, most studies found that there was no overt dystonia manifestation (35). However, in an animal study examining four independent lines of transgenic mice overexpressing the abnormal human torsinA, approximately 40% of the mice presented with abnormal dystonia-like behaviors such as self-clasping and head deviation. Of note, ubiquitin-positive and torsinA-positive perinuclear inclusions were prominently aggregated in the cholinergic neurons of the PPN, whereas no pathologic changes were found in other brain areas, such as the cerebral cortex, hippocampus, striatum, SNpc, and cerebellum (34). Another study has explored the behavior and neurochemical lesions in a dystonia musculorum mutant mouse model. The animal model had severe dystonic movements, motor coordination deficits, and spastic ataxia, which correlated with an increase in acetylcholinesterase activity in the PPN, along with major regions of the basal ganglia (33).

### Dystonia and the change in muscle tone under the intervention of the PPN

Dystonia and the change in muscle tone were observed when the PPN underwent pharmacological lesions and neuromodulation. It is assumed that the cholinergic integrity of the PPN might closely correlate with muscle tone and the development of dystonia. Rats that have underwent bilateral non-specific lesions of the PPN exhibited increased muscle tone and abnormal flexion of the spine and limbs while suspended (36). In another study, monkeys that received unilateral non-specific lesions of the PPN also showed flexed posture, hypokinesia, and rigidity of the limbs contralateral to the lesioned side, and these symptoms gradually improved and became stationary in 2–3 weeks after surgery (37). When the cholinergic cell-specific lesion was performed on the PPN, the primates showed imbalanced muscle tone between the hindlimbs of the lesioned and non-lesioned sides. Additionally, the rigidity of the tail and proximal part of the limb contralateral to the lesioned side also appeared along with the back curvature (12). Moreover, when wild-type human tau was specifically expressed in the cholinergic neurons of the PPN, rats showed dystonia-like behavior of the hindlimb (hindlimb retracted toward body, crossing, and immobility) while suspended (38).

Neuromodulation studies examining PPN have indicated that low-frequency electrical stimulation of the PPN could induce a change in muscle tone, which presented as atonia and hypotonia; this may be due to the change in the neural activity of the cholinergic neurons in the PPN. In de-cerebrated cats, low-frequency electrical stimulation of the PPN suppressed the muscle tone of hindlimbs, and this effect was also elicited through either GABA antagonists or glutamatergic agonist injection into the PPN. Moreover, when the cholinergic muscarinic receptor antagonist was injected into the downstream reticular formation of the PPN, the suppression effect of the PPN stimulation was abolished, which indicates that the cholinergic output from the PPN could modulate the muscle tone through the reticular formation (20). Meanwhile, electrical stimulation of the PPN could also suppress the muscle tone of the neck and forelimb in decerebrated cats, and the suppressive effect was noted to be frequency- and intensity-dependent (39). Furthermore, when topographically analyzing the stimulation sites that induced atonia and hypotonia in the PPN, they were found to agree well-with the dorsoventral distribution of the cholinergic neurons in the PPN (5). However, an early study demonstrated that electrical stimulation of the PPN could produce an excitatory effect on bilateral cervical muscles, and the effect of excitation decreased while the frequency of stimulation increased (40). This finding might be explained by the fact that the PPN could both innervate the excitatory and inhibitory zones of reticular formation, although the specific mechanism still needs to be explored.

Electrical stimulation studies could not fully elucidate the function of the PPN because they were mainly based on the transected animals that lack the descending inhibitory control over the PPN (21). In normal animals, a recent optogenetic study (41) found that short-term photoactivation of the glutamatergic neurons of the PPN could evoke strong and fast motor responses in the bilateral flexor muscles of hindlimbs, whereas the photoactivation of the cholinergic neurons might induce strong and slower motor changes in bilateral extensor muscles. Additionally, long-term photoinhibition of both the glutamatergic and cholinergic neurons increased the extensor burst. In another study (2), the photoactivation of glutamatergic neurons of the PPN was shown to induce long-lasting increases in the muscle tone of flexors such as the biceps motor activity and gait performance. Combined with the former findings, the cholinergic neuropathy of the PPN might cause dystonia and be closely related to flexor muscle hyperexcitation.

With regard to the pathophysiology of dystonia, loss of inhibition at multiple levels of nervous system including the cortex, brainstem and spinal cord and impaired sensory-motor integration have been noted in certain patients (42–44). In particular, the surround inhibition (the decrease of corticospinal excitability of the surround muscles nearby the active muscles) was absent in patients with focal hand dystonia (45). And deficits of the afferent inhibition (the decrease of the motor-evoked potential under a peripheral electrical stimulation to the hand prior to the contralateral motor cortex stimulation) have also been found in patients of dystonia (44, 46). Based on the evidence of dystonia and muscle tone change after the PPN intervention, it is assumed that the cholinergic neuropathy of the PPN might induce the hyperexcitation of flexor muscles through its disinhibition on the reticular formation or its effect on the basal ganglia-cerebello-thalamo-cortical circuit, which in turn contribute to the impairments of surround inhibition and sensory-motor integration.

### Role of the PPN in the modulation of different locomotion patterns

The PPN has also been determined to be involved in the modulation of different patterns of locomotion, and the abnormal locomotion caused by the PPN intervention may be due to dystonia (47). Several studies have explored the effect of PPN on locomotion through *in vivo* electrophysiological recording, neuromodulation, and pharmacological lesions, wherein it was found that the PPN could modulate the motor initiation, duration, rhythm, and speed (2, 4, 41). Through sing-unit activity recording, the change in neuronal activity in the PPN preceded the onset of bilateral limb movements in primates, indicating its role in motor initiation (48). When the motor processes were further specified and correlated with the neural activity of the PPN, it was found that neurons in the PPN were preferentially related to the onset and termination of motor episodes (3).

Interventional experiments have also elucidated that electrical stimulation of the PPN could elicit machine-like coordinated locomotion in decerebrated animals (5, 49). Recent optogenetic studies have shown that activation of the cholinergic neurons of the PPN could slow or terminate the ongoing locomotion, while inhibition might increase the step cycle (4, 41). However, it remains unclear whether photoactivation of the glutamatergic neurons could elicit low-speed locomotion and explorative behavior (2, 4). There is also evidence that activation of the glutamatergic neurons of the PPN might rescue the severe locomotor deficit and produce high-quality locomotion in mouse models of parkinsonism (50). Moreover, non-specific pharmacologic studies on the PPN showed that the spontaneous or drug-induced locomotion was unaffected (51–53). Nevertheless, excitotoxic lesions of the PPN might impair fine motor control and increase the incidence of oral motor behavior under intra-putamen injection of amphetamine in rats (54, 55).

## Pathologic change of the PPN in patients with dystonia

According to associated features, dystonia can be classified into isolated dystonia (dystonia is the only motor feature) and combined dystonia (dystonia combined with other movement disorders) (7). Both types of dystonia are featured by the deficits of network involving the basal ganglia-cerebello-thalamo-cortical circuit (56). And pathologic changes in the PPN are found in both types of dystonia.

### Pathologic changes of the PPN in isolated dystonia

At present, no consistent or specific neuropathologic changes have been found in postmortem studies of patients with isolated dystonia (58). Several autopsy reports of patients with isolated dystonia showed that the pathologic changes of the PPN include the presence of neurofibrillary tangles (NFTs), cholinergic neural loss, and perinuclear inclusions (Table 1). In the autopsy report of a 29-year-old male patient with dystonia musculorum deformans, NFTs were found in the locus coeruleus (LC), SNpc, PPN, and dorsal raphe nucleus. The patient presented with early-onset dystonia, mainly affecting the right foot, both hands and head (10). In another 68-year-old male patient with Meige syndrome (blepharospasm combined with oromandibular dystonia), the significant neural loss was observed in the SNpc, dorsal raphe, PPN, and LC along with astrocytic proliferation (10). Besides NFTs and neural loss found in the PPN, the cholinergic pathology of the PPN has also been found correlated with dystonia in some studies (8, 9). In a study that enrolled four patients with a GAG deletion of the dystonia gene *DYT1*, ubiquitin-positive and torsinA-positive perinuclear inclusions were found to be aggregated in cholinergic and other neurons of the PPN, cuneiform nucleus, and periaqueductal gray (8). Although these results were not confirmed by a recent study (58), absent or reduced choline acetyltransferase staining was confirmed in all six available PPN samples in another recent study examining eight patients with cervical dystonia (9); this, together with the finding that the diffusion tensor imaging of the PPN was identical between patients and controls, suggests that a functional cholinergic deficit might be involved in cervical dystonia (9).

**Table 1:** Summary of pedunculopontine nucleus involvement in dystonia-related autopsy reports.

**Report**	**Diagnosis**	**Age at onset/death, yr**	**Family history**	**Gender**	**Genotype**	**Phenotype**	**PPN pathology**	**Other neuropathology**
**Isolated dystonia**								
Zweig et al. (10)	DMD	14/29	Likely	M	-	Dystonic posturing of right foot and both hands, mild head forward, hypomimia	Occasional NFTs	Neural loss in LC; NFTs in LC, SNpc and DRN
	Meige syndrome	33/68	-	M	-	Oromandibular dystonia, occasional blepharospasm, Retrocollis	Neural loss	Neural loss in SNpc, DRN and LC and CSRN; astrocytic proliferation and extracellular neuromelanin pigment in SN and LC; infrequent NFTs in SN
McNaught et al. (8)	DMD	5/83	Yes	F	DYT1-GAG deletion	No details	Perinuclear ubiquitin and torsinA-positive inclusions in cholinergic neurons	Ubiquitin and torsinA-positive inclusions in RF, PAG, SC, CN and griseum centrale mesencephali; Tau and ubiquitin-positive inclusions in SNpc and LC
	Focal non-progressive dystonia	19/86	Yes	M	DYT1-GAG deletion			
	DMD	10/78	Yes	M	DYT1-GAG deletion			
	Generalized dystonia	8/33	Likely	M	DYT1-GAG deletion			
Mente et al. **(****9****)**	CD	47/52	-	F	-	Left torticollis	Cholinergic neural loss	None
	CD	40/65	-	F	-	Right laterocollis		
	CD	Unknown/70	-	M	-	No details		
	CD	53/75	–	F	-	No details		
	CD	Unknown/78	-	F	-	No details		
	CD	Unknown/81	-	F	-	Dystonic head tremor		
	CD	41/84	-	F	-	Left laterocollis		
	CD	Unknown/85	-	F	-	Dystonic head tremor		
**Combined dystonia**					-			
Sasaki et al. (57)	Early onset Parkinson's disease	33/70	-	F	Parkin-homozygous exon 3 deletion	Juvenile parkinsonism, choreic dyskinesia of limb and trunk, gait disturbance, bilateral extension of big toes	Basophilic, α-synuclein and ubiquitin-positive inclusions	Melanin-containing neurons loss in SNpc and LC; extraneuronal melanin pigments in LC

CD, cervical dystonia; CN, cuneiform nucleus; CSRN, central superior raphe nucleus; DMD, dystonia musculorum deformans; DRN, dorsal raphe nucleus; LC, locus ceruleus; NFTs, neurofibrillary tangles; PAG, periaqueductal gray; PPN, pedunculopontine nucleus; RF, reticular formation; SC, superior colliculus; SNpc, substantia nigra pars compacta.

### Pathologic change of the PPN in dystonia combined with parkinsonism

Dystonia combined with parkinsonism has been determined as the most common type of combined dystonia. Within PD, the prevalence of dystonia could be more than 30% if the onset of parkinsonism is before the age of 40 (59). Various types of dystonia can co-occur with parkinsonism, including blepharospasm, camptocormia, Pisa syndrome, anterocollis, and limb dystonia. Among them, lower limb dystonia is relatively common (60). Patients with lower limb dystonia mainly present with ankle inversion, hyperextension of the big toe, and flexion of the other toes, which is also known as “striatal foot.” The striatal foot might appear due to dystonia, rigidity, and inappropriate contraction of the foot muscle, and the dystonic, disinhibited foot grasp might also induce the freezing of gait (47). At present, DBS of the PPN showed beneficial effect on improving gait freezing (61), which may be attributed to its effect on the locomotion along with foot dystonia.

Only a few studies mention the relationship between the presentation of dystonia combined with parkinsonism and the pathological changes in the brain. The available studies show that among the patients with dystonia combined with parkinsonism, there are NFTs and inclusions in the PPN, and the pathological changes in the PPN are more related to the lower limb (57). Patients of PD with parkin gene mutation commonly have dystonia as their initial symptom, which is known to often affect the lower limb (59). In the autopsy report of a 33-year-old female patient with PD with a homozygous exon 3 deletion in the *parkin* gene, basophilic inclusions were occasionally found in the PPN, and ubiquitin-positive and α-synuclein-positive inclusions were also observed (Table 1). The dystonia of the patients mainly presented as a bilateral extension of large toes (57). Apart from the autopsy studies, DBS of the PPN also showed marked efficacy in alleviating axial dystonia in patients with PD, which might reflect the relationship between PPN and dystonia from the therapeutic aspect. Shih et al. (11) reported that DBS of the contralateral PPN to the bending side could improve not only gait but also dystonia in a 62-year-old female patient with PD combined with Pisa syndrome. Additionally, Ricciardi et al. (62) found that DBS on the ipsilateral PPN to the bending side could also provide short-term benefits on PD associated with Pisa syndrome. Although patients with PD often develop Pisa syndrome away from the side with predominant symptoms, the efficacy of either side of the PPN DBS might be due to the bilateral modulative effect of the PPN on axial muscle tone (63).

## Potential mechanisms underlying PPN pathology on the development of dystonia

Dystonia is known to be a network disorder characterized by the involvement of the basal ganglia-cerebello-thalamo-cortical circuit. The pathology in the basal ganglia and cerebellum has often been observed in patient and animal models of dystonia (56, 64). In addition, the imbalance between the striatal dopamine and acetylcholine systems has commonly been regarded as the key mechanism for the development of dystonia (65). Primates and humans could develop dystonia under the treatment of acetylcholine agonists and dopaminergic antagonists (66, 67). Most types of dystonia could be effectively treated with anticholinergic therapy (68). Moreover, in the striatum of the Dyt1 mouse model, the dopamine D2 receptor agonist could induce paradoxical excitation of the cholinergic interneurons, rather than the physiological inhibition, leading to the over release of acetylcholine and dysfunction of the striatum (69). As mentioned above, the PPN has various connections with both the basal ganglia and cerebellum. The PPN might be primarily affected in isolated dystonia whereas secondarily affected in PD combined with dystonia. The primary deficit of the PPN has been determined in patient and animal models of the Dyt1 mutation, which mainly presented as cholinergic pathology and was involved in the integration and output of the motor information (8, 34). On the basis of the directly modulative effect the PPN on the medium-sized spiny neurons and interneurons in the striatum mentioned above, the cholinergic loss of the PPN might ascendingly contribute to the dysfunction of the central selection, the neural plasticity, and the imbalance of the dopamine and acetylcholine in the striatum (19). In addition, the cholinergic lesion of the PPN were significantly associated with loss and morphologic changes of nigral dopaminergic neurons, which indirectly affects the dopamine release in the striatum through the nigra-striatum pathway (70). Meanwhile, patients with dystonia have been reported as hypoactivity at the cerebellar cholinergic terminals at lobules VI and VII that response for the cerebellar sensorimotor projections to the cortex and working memory, which reflects that the abnormal cholinergic input form the PPN to the cerebellum might be related to the impaired sensorimotor integration and cognitive functions in patients of dystonia (15). Moreover, the cholinergic lesion of the PPN could also descendingly contribute to the disinhibition of the motor neurons and interneurons in the spinal cord, leading to muscle hyperexcitaion and loss of surround inhibition (21) (Figure 1B).

In dystonia combined with parkinsonism, the PPN might be affected secondary to the pathological change of the basal ganglia-cerebello-thalamo-cortical circuit. PD is featured by the neurodegeneration of the dopaminergic neurons in the SNpc. The deficit of the dopamine release in the striatum results in the overactivity of the indirect pathway. The overactivity of the GPi and SNpr could suppress the neural activity of the thalamus, leading to decreased activity of the cortex and presence of parkinsonism (71). On the other hand, it could decrease the neural activity of cholinergic and glutamatergic neurons in the PPN, which directly induces the disinhibition of the reticular formation and spinal cord or indirectly affects the function of the striatum, thalamus and cortex (1). Moreover, in a PD rat model under intra-nigral lesion with lactacystin, alpha-synuclein aggregation might trigger both PPN cholinergic and non-cholinergic neuron loss via neuronophagia (72). The PPN neuron loss triggered by the pathological change of dopaminergic neurons in the SNpc might further contribute to the dystonia manifestations in PD patients such as striatal foot and Pisa syndrome as mentioned above. Together with the neurodegeneration of dopaminergic neurons in the SNpc, the striatal cholinergic impairment might also affect the PPN and induce dystonia. Striatal cholinergic dysregulation was noted after neonatal decrease in X-Linked dystonia parkinsonism-related TAF1 isoforms in mice (73). And loss of striatal interneurons was also found in rapid-onset dystonia-parkinsonism mice (74). In summary, the role of the PPN in the dystonia combined with parkinsonism is complicated and its effects on the basal ganglia, cerebellum and spinal cord are under further exploration (Figure 1C).

Regarding the non-motor manifestations of dystonia, patients of primary dystonia had deficits on a broad range of cognitive domains including global cognitive function, attention, memory and conceptualization (75). In more detail, dystonia patients presented cognitive impairments related to the executive dysfunctions such as set-shifting, category fluency and verbal learning, and impaired time-based prospective memory was also found in patients of cervical dystonia, reflecting the potential dysfunctions in the prefrontal cortex and basal ganglia (76, 77). The PPN might be involved in the cognitive functions through its connections with the cortex, thalamus (especially the thalamic centromedian-parafascicular complex), basal ganglia and cerebellum as mentioned above, and cognitive impairments mimicked non-motor manifestations of dystonia could appeared following the PPN lesion. In detail, different types of learning (mnemonic motor task related learning, reversal learning and stimulus-reward related learning) and attentional performance were impaired following the PPN lesion in rats (13, 52, 78, 79). In addition, dystonia patients have been reported poor sleep quality and disturbed sleep architecture, and these impairments of sleep might not due to the persistence of muscle activity (80). In particular, certain dystonia patients showed increased latency of rapid eye movement (REM) sleep and reduced REM duration (81). It is reported that the PPTg had cholinergic projections to the gigantocellular tegmental field, which might participate in the induction and maintenance of normal REM sleep and cortical activity (6). And rat studies showed that lesion of the PPN could disturb sleep/wake state transitions and reduce the REM sleep duration under hypoxic conditions, which might be in favor of the involvement of the PPN in the sleep disturbance of dystonia (14, 82). Moreover, from the therapeutic aspect, bilateral low-frequency DBS of the PPN improved simple reaction times without a warning cue in PD patients, indicating its beneficial effect on the attentional processing (83). And improved executive functions and working memory were also noted after low-frequency DBS of bilateral PPN for PD patients (84, 85). Furthermore, bilateral low-frequency PPN DBS also produced marked cognitive improvement associated with a significant increase in cortical metabolism in both prefrontal areas and mono-lateral ventral striatum (86). Regarding the sleep disturbance, unilateral low-frequency DBS of the PPN significantly doubled the duration of REM sleep in PD patients through increasing REM sleep episodes, indicating that the PPN DBS might affect the transitioning between sleep states (87). While no improvement of cognitive, psychiatric or emotional functions for PD patients underwent PPN DBS were also found in other studies (88, 89), theses above clinical evidences are in favor of the PPN involving in the non-motor manifestations of movement disorders, and whether DBS of the PPN could contribute to the improvement of non-motor manifestations in the dystonia still need further exploration.

## Conclusion

Preclinical and clinical evidences have revealed the involvement of the PPN in the development of dystonia. The dense anatomical connectome between the PPN and dystonia-related basal ganglia-cerebello-thalamo-cortical circuit lays the foundation for the contributions of the PPN to both motor and non-motor manifestations of dystonia. Of note, the cholinergic pathology of the PPN might affect the balance of striatal dopamine and acetylcholine systems and mimic the pathophysiological processing of dystonia. Different neural populations of the PPN might have distinct effects on motor and non-motor functions, and their roles in the development and treatment of dystonia requires further exploration to provide evidence for targeting the PPN for the treatment of dystonia in the future.

## Author contributions

J-hS: design and writing. Y-wH: visualization. YY: resources. R-yL and L-xL: revising. FT: design and revising. L-jJ: reviewing, supervision, and editing of final version of the manuscript. All authors contributed to the article and approved the submitted version.

## Funding

This work was supported by Medical Innovation Project of Shanghai Science and Technology Commission (20Y11906000), Outstanding academic leader of Shanghai Science and Technology Commission (20XD1403400), National Natural Science Foundation of China (81971074), and Clinical Science and Technology Innovation project of Shanghai Shen-kang Hospital Development Center (SHDC12020119).

## Conflict of interest

The authors declare that the research was conducted in the absence of any commercial or financial relationships that could be construed as a potential conflict of interest.

## Publisher's note

All claims expressed in this article are solely those of the authors and do not necessarily represent those of their affiliated organizations, or those of the publisher, the editors and the reviewers. Any product that may be evaluated in this article, or claim that may be made by its manufacturer, is not guaranteed or endorsed by the publisher.
